# 

**Published:** 1905-09-02

**Authors:** 


**DEATH.**

**Notices of Births, Deaths, and Marriages** are inserted in this column at a charge of 2s. 6d. for an announcement not exceeding four lines.

Mears—On the 23rd ult., the wife of Dr. F. C. Mears, North Shields. (2550)

**NOTICE TO CORRESPONDENTS.**

All MSS., Letters, Books for Review, and other matters intended for the Editor should be addressed The Editor, "The Hospital," The Hospital Buildings, 28 & 29 Southampton Street, Strand, W.C.

The Editor cannot undertake to return rejected MS., even when accompanied by stamped directed envelope.

**Notice to Advertisers and Subscribers.**

All Advertisements, Orders for Copies of The Hospital, and Business Communications should be addressed to **THE MANAGER (Not to the Editor),** The Hospital, 28 & 29 Southampton Street, Strand, London, W.C.

The Cost of Subscription, post free, is at follows :—Per week, 3½d. ; per quarter, 3s. 3d. ; per half-year, 6s. 6d. ; per annum, 13s. Foreign and Colonial :—Per annum, 19s. 6d., payable, in all cases, in advance.

**NOTICE.—Vol. XXXVII. of "The Hospital" October 1904 to March 1905), in handsome cloth binding, will shortly be on sale at the office of this Journal, price 10s. 6d. Cases for binding the Numbers contained in this Volume 2s. Index to Vol. XXXVII., 3½d., post free.**

**The Monthly Part for September is now ready. Price 1s., by post, 1s. 3d.**

### Medical Appointments.

Thomas Cooke, L.R.C.P.Edin., M.R.C.S.Eng., reappointed Medical Officer of Health for the Hurst Urban District Council. Mary Kidd, M.B.Lond., Junior Resident Medical Officer to the Canning Town Medical Mission, London, E. C. P. Skrimpshire, M.R.C.S.Eng., L.S.A., Certifying Surgeon under the Factory and Workshop Act for the Blaenavon District of the County of Monmouth. Alexander Dey Thompson, M.B., Ch.B.Glasg., Senior Assistant Medical Officer to the Monmouthshire Asylum, Abergavenny.

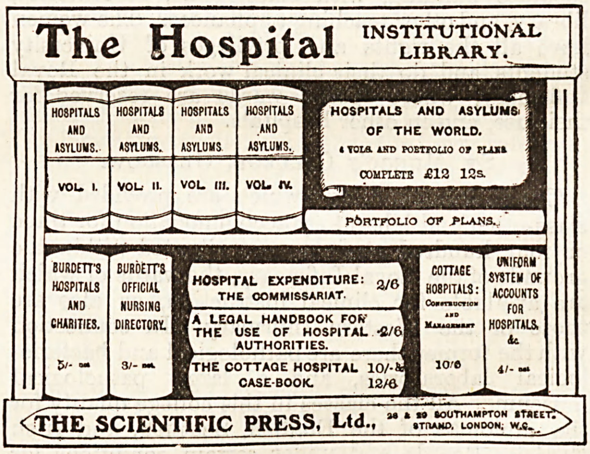

THE BEST NATURAL APERIENT WATER.

**Hunyadi János**

**For LIVER COMPLAINTS, OBESITY, &c.**

The "VIENNA MEDICAL PRESS" says:—

**"Hunyadi János may be regarded as a specific for obesity.**

Average Dose.—*A wineglassful before breakfast, either pure or diluted with a similar quantity of hot or cold water.*

**CAUTION.**—Note the name "Hunyadi János," the signature of the Proprietor, ANDREAS SAXLEHNER, and the Medallion, on the Red Centre Part of the Label. [3

### Useful Handbooks for Mothers and Nurses.

### MOTHER'S HELP AND GUIDE TO THE DOMESTIC MANAGEMENT OF HER CHILDREN.

By Dr. Braidwood. 2/- post free.

A handy and intelligible manual on the treatment of the Child from earliest infancy. Mothers and Nurses will find it of great assistance in the General Management of the Nursery.

### THE COMMONWEALTH OF THE BODY.

By Dr. Hawkins Ambler. 1/- post free.

A series of interesting papers on Physiology and the laws of health. Simply and clearly written especially for young people.

### SICK NURSING AT HOME.

By L. G. Moberley. 1/- post free.

A valuable little handbook which cannot fail to be of real practical use to those who are obliged to undertake nursing in their own homes, and who for one reason or another are unable to obtain trained help.

### SIMPLE SANITATION:
### The Practical Application of the Laws of Health to Small Dwellings.

By M. Loane. 1/- net, 1/2 post free.

This work should prove of great practical assistance in the household, as it contains Chapters on Sanitary Legislation, Water, Air and Ventilation, Drainage and Disposal of Refuse, Household Management, Personal Hygiene, Disinfectants, &c.

### TENDENCIES TO CONSUMPTION:
### How to Counteract them.

By Dr. J. D. E. Mortimer. 2/6 post free.

A work of the utmost value to all who have the care of a delicate child or person with a hereditary tendency to Consumption. The information, if intelligently followed, would do much to prevent the development of this disease.

### DIET IN SICKNESS AND IN HEALTH.

By Mrs. Ernest Hort. 3/6 post free.

A useful help to those who are sick, and to those who have to nurse, feed, and prescribe for the sick. It will also aid the healthy to preserve health.

### THE ART OF FEEDING THE INVALID.

NEW EDITION. With Special Chapter on Food for Consumptives. 1/6 post free.

This is an invaluable handbook in the Sick-room, the Household, and to all who have the care of Invalids and Convalescents. It contains chapters on Foods in Indigestion, in Corpulence, in Constipation and Diarrhœa, in Gout, in Liver and Kidney Diseases, in Consumption, in Convalescence and Chronic Illness, &c., with a large number of nourishing and appetising recipes.

### CLEANLINESS IN CHILDREN.

By Rose Petty. 2d. post free.

A handbook for the use of all who have the care of young children.

**The Scientific Press, Ltd.,** 28 & 29 Southampton Street, Strand, London, W.C.

Complete Catalogue Post free on application.

